# Galectin-9 Ameliorates Con A-Induced Hepatitis by Inducing CD4^+^CD25^low/int^ Effector T-Cell Apoptosis and Increasing Regulatory T Cell Number

**DOI:** 10.1371/journal.pone.0048379

**Published:** 2012-10-31

**Authors:** Kun Lv, Yingying Zhang, Mengying Zhang, Min Zhong, Qifeng Suo

**Affiliations:** 1 Central Laboratory of Yijishan Hospital, Wannan Medical College, Wuhu, People’s Republic of China; 2 Laboratory Medcine of Yijishan Hospital, Wannan Medical College, Wuhu, People’s Republic of China; University of Pittsburgh, United States of America

## Abstract

**Background:**

T cell-mediated liver damage is a key event in the pathogenesis of many chronic human liver diseases, such as liver transplant rejection, primary biliary cirrhosis, and sclerosing cholangitis. We and other groups have previously reported that galectin-9, one of the β-galactoside binding animal lectins, might be potentially useful in the treatment of T cell-mediated diseases. To evaluate the direct effect of galectin-9 on hepatitis induced by concanavalin A (Con A) administration in mice and to clarify the mechanisms involved, we administered galectin-9 into mice, and evaluated its therapeutic effect on Con A-induced hepatitis.

**Methodology/Principal Findings:**

Galectin-9 was administrated i.v. to Balb/c mice 30 min before Con A injection. Compared with no treatment, galectin-9 pretreatment significantly reduced serum ALT and AST levels and improved liver histopathology, suggesting an ameliorated hepatitis. This therapeutic effect was not only attributable to a blunted Th1 immune response, but also to an increased number in regulatory T cells, as reflected in a significantly increased apoptosis of CD4^+^CD25^low/int^ effector T cells and in reduced proinflammatory cytokine levels.

**Conclusion/Significance:**

Our findings constitute the first preclinical data indicating that interfering with TIM-3/galectin-9 signaling *in vivo* could ameliorate Con A-induced hepatitis. This strategy may represent a new therapeutic approach in treating human diseases involving T cell activation.

## Introduction

Acute and chronic liver diseases are still major health problems caused by various etiologies. Immune-mediated mechanisms have a central role in autoimmune and viral hepatitis, and in determining disease outcomes [1]–[4]. Despite the availability of advanced treatments, a high percentage of individuals still fail to respond to conventional methods of treatment, and in some, liver transplantation is ultimately required. Therefore, a better understanding of immune mechanisms underlying hepatitis is needed for the generation of more effective therapeutic strategies against the disease. Current evidence suggests that inhibiting the over-activated immune response or directly preventing liver cell damage may have a beneficial effect on liver diseases.

Recently, a new hepatitis model has been developed, in which concanavalin A (Con A) injection into mice leads to a dose-dependent liver injury. That T cell activation is a crucial factor in this hepatitis model is shown by the resistance of severe combined immunodeficiency disorder mice, which lack immunocompetent T and B lymphocytes, to Con A-induced hepatitis [5], [6]. Con A-induced hepatitis is accompanied by an increase in the serum concentration of several proinflammatory cytokines, including tumor necrosis factor-α (TNF-α), interleukin-6 (IL-6), interferon-γ (IFN-γ) and interleukin-1 (IL-1) [7], [8] which contribute to the development of hepatitis [5], [9]. Furthermore, pretreatment with anti–IFN-γ or anti–TNF-α monoclonal antibodies (mAbs), or IFN-γ gene ablation confers protection against Con A-induced hepatitis, indicating that Th1-dependent cytokines are also involved [9]–[12].

Galectin-9, one of the β-galactoside binding animal lectins belonging to the galectin family, induces apoptosis of eosinophils, cancer cells, and T cells [13]–[16]. Galectin-9 preferentially induces apoptosis of activated CD4^+^ T cells through Ca^+^ influx-calpain-caspase1 pathway [17]. Zhu et al. have demonstrated that galectin-9 is a ligand of T cell immunoglobulin- and mucin domain-containing molecule 3 (TIM-3) that was expressed selectively on terminally differentiated Th1 cells [17], Th17, regulatory T cells (Tregs) [18], and that galectin-9 induces apoptosis of TIM-3-expressing cells *in vitro* and *in vivo* [19]. Valerie et al. have found that TIM-3/galectin-9 pathway regulates Th1 immunity through promotion of CD11b^+^Ly-6G^+^ myeloid cells formation [20]. In fact, exogenous administration of galectin-9 ameliorates experimental allergic encephalitis, an autoimmune disease of the central nervous system [19]. Furthermore, galectin-9 exhibits an anti-inflammatory role in LPS-induced inflammation [21] and in experimental allergic conjunctivitis (EAC) in mice [22]. More recently it has been shown that galectin-9 ameliorates symptoms in a mouse collagen-induced arthritis (CIA) model and reduces HSV induced lesions, by regulating the T cell response [23], [24]. Our previous study has indicated that galectin-9 administration effectively ameliorates CVB3-induced myocarditis by promoting the proliferation of T regulatory cells and the activation of Th2 cells [25]. All together, these data suggest that galectin-9 might be potentially useful in the treatment of T cell mediated diseases.

The aims of this study were to investigate the effect of galectin-9 on Con A-induced hepatitis in mice and to clarify the mechanisms involved.

## Results

### Role of T Cell Subsets in Con A-induced Hepatitis

**Figure 1:**
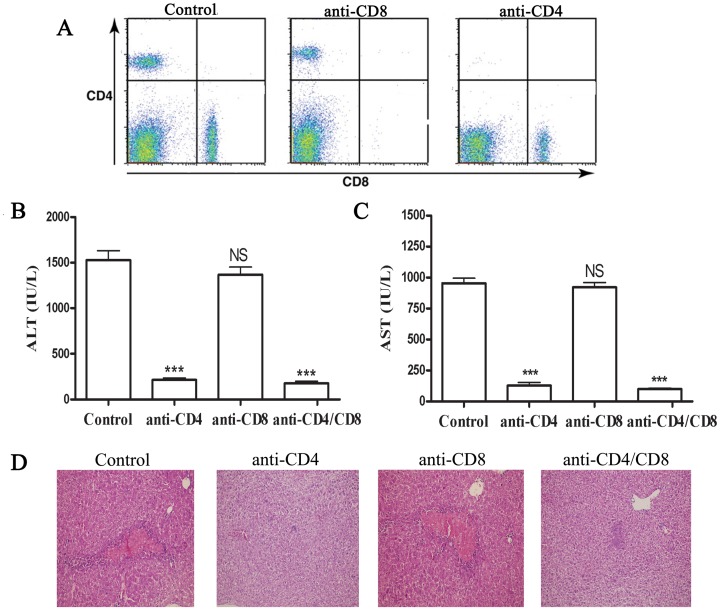
T cells and Con A-induced hepatitis. Isotype, CD4 and/or CD8 mAbs (100 µg per mouse) was administrated i.p. to Balb/c mice (n = 8 per group) 24h before Con A injection (20 mg kg^–1^). The effect of anti-CD4 orCD-8 antibodies administration on depleting these cells in the liver of mice prior injecting Con A was determined by FACS. Representative results were shown in (A). Sera were collected 24 h after Con A injection. Serum ALT (B) and AST (C) levels were measured. The results were presented as the mean ± SD of three separate experiments. ***, p<0.001 vs control. (D) The livers were removed 24 h later. Paraffin sections were stained with hematoxylin and eosin (H&E) staining. Representative liver sections were shown for each group, original magnification: ×200.

In vivo depletion of CD4- and CD8-specific T cells is a means of studying the role of these subpopulations in the initiation and effector phases of particular in vivo immune responses. As shown in Fig. 1A, administration of anti-CD4 or CD8 antibodies effectively depleted these cells in the liver of mice prior injecting Con A. Administration of Con A intravenously in mice resulted in significant liver injury and elevation of serum ALT and AST levels in mice (Fig. 1B and 1C). Furthermore, histological analysis of the liver in mice injected with Con A revealed massive liver apoptosis and necrosis, with accompanying hemorrhage (Fig. 1D). Induction of liver injury requires CD4^+^ T cells, as depletion of these cells significantly reduced serum ALT and AST levels compared with isotype control group (Fig. 1B and 1C). Meanwhile, in anti-CD4 mAb treated mice, the liver tissue showed normal histology and significantly less apoptosis and necrosis compared with isotype control group (Fig. 1D). Depletion of CD8^+^ T cells did not attenuate liver injury; however, depletion of both CD4^+^ and CD8^+^ T cell reduced liver injury to a greater extent than just depleting CD4^+^ T cells alone (Fig. 1B, 1C and 1D).

### TIM-3 Signaling May Serve to Regulate Con A-induced Hepatitis

**Figure 2:**
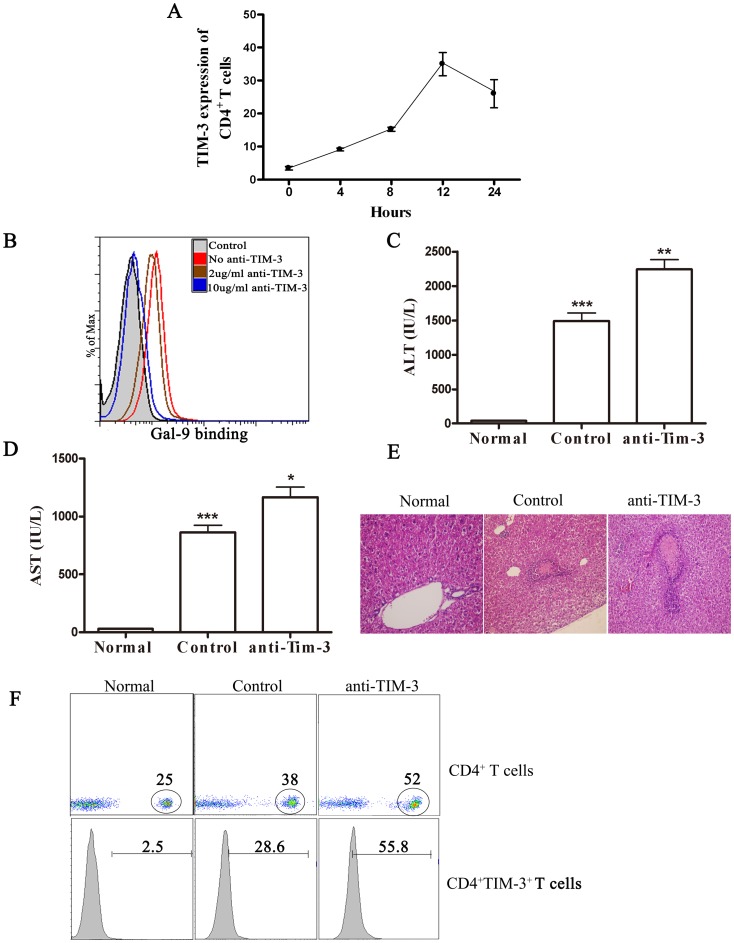
Effect of TIM-3 blockade on liver inflammation and CD4^+^ T cell immune response. Isotype or TIM-3 mAb (100 µg per mouse) was administrated i.v. to Balb/c mice (n = 8 per group) 30 min before Con A injection (20 mg kg^–1^). (A) TIM-3 expression of CD4^+^ T cell in spleen was detected 24 h following Con A injection. (B) The effect of anti-TIM-3 on the binding of galectin-9 to mouse Th1 cells. CD4^+^ T cells were purified from splenocytes of normal mice by negative selection with magnetic beads. Cells (1×10^6^ cells/ml) were cultured for 5 d with phytohemagglutinin (1 mg/ml) and IL-2 (8 ng/ml) in polarizing conditions: IL-12 (2 ng/ml) plus antibody to IL-4 (anti-IL-4; 100 ng/ml; MP4-25D2); Cells (5×10^5^ cells/ml) were collected and incubated for 1 h at 4°C with biotinylated galectin-9 in the presence or absence of increasing anti-TIM-3 (2 ug/ml or 10 ug/ml). Cells were then incubated for 45 min at 4°C with fluorescein isothiocyanate–conjugated streptavidin, were washed and were analyzed by FACS. Serum ALT (C) and AST (D) levels were measured 24 h after Con A injection. The results were presented as the mean ± SD of three separate experiments. **, p<0.01; *, p<0.05. (E) The livers were removed 24 h later. Paraffin sections were stained with hematoxylin and eosin (H&E) staining. Representative liver sections were shown for each group, original magnification: ×200. (F) Percentages and phenotype (surface TIM-3) of CD4^+^ T cells in spleen of mice are shown. Normal, normal mice; Control, PBS treatment in Con A-treated mice; anti-TIM-3, anti-TIM-3 mAb pretreatment in Con A-treated mice.

As a prelude to exploring the value of manipulating TIM-3/galectin-9 interaction to influence the outcome of Con A-induced hepatitis, mice were injected with Con A and the expression pattern of TIM-3 on CD4^+^ T cells was measured at various times in the spleen. We found that few CD4^+^ T cells expressed TIM-3 in naive animals. The number of TIM-3^+^CD4^+^ T cells increased significantly at 4 hours, peaked at 12 hours and declined markedly 24 hours later (Fig. 2A). To further confirm whether TIM-3 signaling has a role in Con A-induced liver inflammation, the mice were pretreated by anti-TIM-3 mAb to neutralize TIM-3. As shown in Fig. 2B, anti-TIM-3 mAb could effectively prevent the binding of biotinylated Gal-9 to Tim3 expressing Th1 cells. As shown in Fig. 2C–E, after TIM-3 neutralization, the ALT, AST level and the liver necrosis were significantly increased. Furthermore, the ratio of CD4^+^ T cells was significantly greater in the spleen from anti-TIM-3-treated mice than in controls (Fig. 2F). With regard to phenotype, the frequency of CD4^+^ T cells that were TIM-3^+^ was significantly higher in the anti-TIM-3 mAb group than in controls. The reason for this observation is not clear, but conceivably it could have reflected the finding that the magnitude of CD4^+^ T cell responses was higher in the anti-TIM-3 mAb-treated animals than in control mice. These data suggest that TIM-3 signaling is involved in Con A-induced hepatitis.

### Galectin-9 Administration Ameliorated Con A-induced Hepatitis

**Figure 3:**
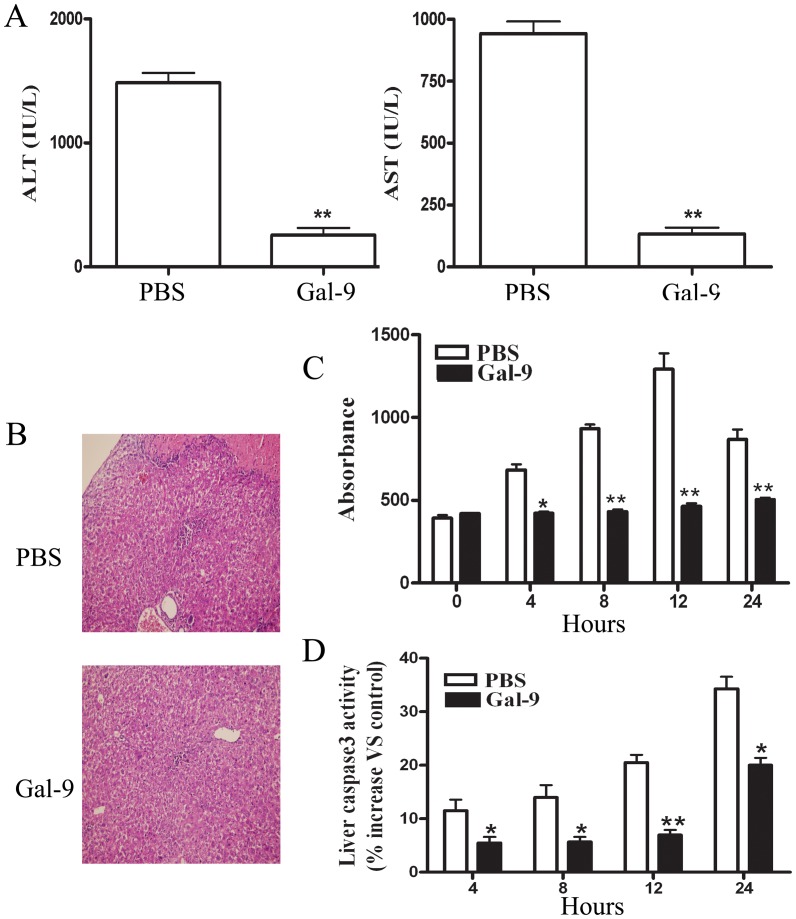
Effect of galectin-9 treatment on Con A-induced hepatitis. Galectin-9 (100 µg per mouse) or PBS was administrated i.v. to Balb/c mice (n = 8 per group) 30 min before Con A injection (20 mg kg^–1^). (A) Serum ALT and AST levels were measured 24 h after Con A injection. (B) The livers were removed 24 h later. Paraffin sections were stained with hematoxylin and eosin (H&E). Representative liver sections were shown for each group, original magnificatios: ×200. (C) Liver DNA fragmentation was monitored by assessing histone ELISA, with or without pretreatment with galectin-9. (D) Time course of liver caspase 3–like activity are shown. The results (A, C, D) were presented as the mean ± SD of three separate experiments. **, p<0.01; *, p<0.05 vs PBS treatment; PBS, PBS treatment in Con A-treated mice; Gal-9, galectin-9 pretreatment in Con A-treated mice. Similar data presentation will appear in the subsequent figures.

Galectin-9 was shown to be the endogenous ligand of TIM-3 and that signaling via galectin-9, at least in some T cell subsets, may cause T cells to undergo apoptosis. For this reason, we investigated whether galectin-9 administration protects mice from Con A-induced hepatitis. As shown in Fig. 3A, serum ALT and AST levels were significantly decreased in mice administered with galectin-9 compared with control mice. Consistently, histological analysis of liver sections revealed that infiltrated mononuclear cells and massive necrosis with cytoplasmic swelling of most surviving hepatocytes were found 24 hours after Con A administration. Pretreatment with galectin-9 reduced the extent of liver damage, as evident in the fewer inflammation and limited necrotic lesions (Fig. 3B). Using an ELISA kit that specifically detects histone-associated DNA fragments, liver DNA fragmentation was detected as early as 4 hours after Con A administration (Fig. 3C). Maximal DNA fragmentation occurred at 12 hours and remained significantly higher than basal values until 24 hours. As shown in Fig. 3C, galectin-9 pretreatment almost completely prevented the Con A–induced liver DNA fragmentation.

In addition, Con A administration resulted in a time-dependent increase in liver caspase 3–like activity, suggesting that hepatocyte death depends on caspase cascade activation (Fig. 3D). Galectin-9 pretreatment significantly, but not completely, reduced liver caspase 3–like activity (Fig. 3D).

### Galectin-9 Administration Significantly Reduced CD4^+^ T Cell Infiltration and Pro-inflammatory Cytokines Production

**Figure 4:**
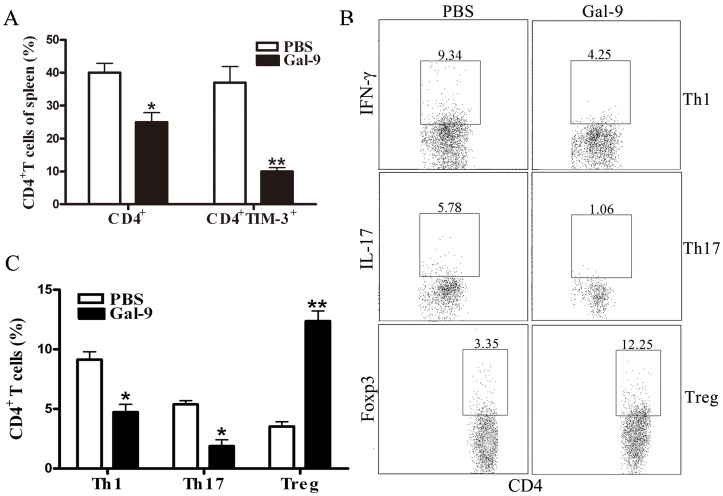
Effect of galectin-9 treatment on cellular infiltration in spleen of Con A treated mice. Galectin-9 (100 µg per mouse) or PBS was administrated i.v. to Balb/c mice (n = 8 per group) 30 min before Con A injection (20 mg kg^–1^). The splenocytes were isolated 24 h later. Percentages and phenotype (surface TIM-3) of CD4^+^ T cells in spleen of mice are shown in (A). (B) The frequencies of Th1, Th17 and Treg subsets were detected by FACS. Statistically significant differences were indicated in (C). The results (A, C) were presented as the mean ± SD of three separate experiments. **, p<0.01; *, p<0.05 vs PBS treatment.

To clarify whether galectin-9 modulates the balance of CD4^+^ T immune response, we determined the number of Th1, Th2, and Tregs in the spleen. As shown in Fig. 4A, the ratio of CD4^+^ T cells was far lower in the spleen from galectin-9-pretreated mice than in controls. Meanwhile, TIM-3 expression was significantly reduced in the CD4^+^ T cells of the galectin-9-pretreated mice than in controls. Furthermore, galectin-9 administration significantly decreased the number of Th1 cells and Th17 cells, but significantly increased Tregs in spleen (Fig. 4B and C).

**Figure 5:**
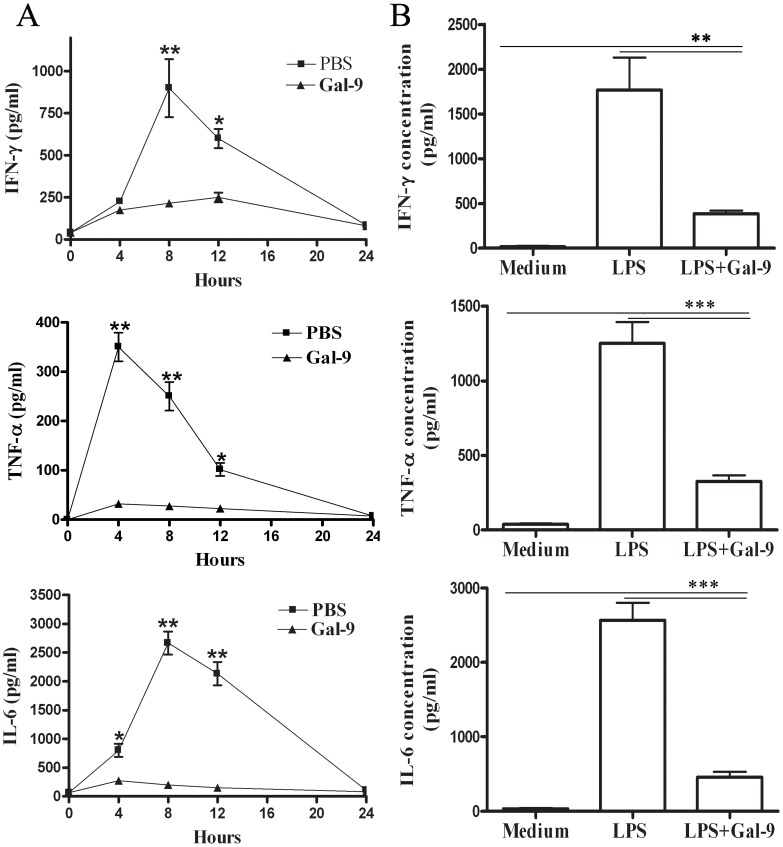
Effect of galectin-9 treatment on inflammatory cytokines secretion *in vitro* and *in vivo*. (**A**) Time course of IFN-γ, TNF-α and IL-6 release into serum after Con A injection, with or without galectin-9 (100 µg per mouse) administered i.v. for 30 minutes before Con A injection. Serum cytokine levels were determined using ELISA-assay kits. Data points represent the mean ± SD for 8 animals killed at each point. **, p<0.01; *, p<0.05 vs galectin-9 pretreated mice. (B) Spleen macrophages (1×10^6^ cells/mL) were incubated for 24 hours with 100 ng/mL LPS, with or without galectin-9 treatment (10 µg/ml). Aliquots of supernatant were then collected and stored at −80°C until assayed. IFN-γ, TNF-α and IL-6 concentrations were measured by using specific ELISA kits. The results were presented as the mean ± SD of three separate experiments. ***, p<0.001; **, p<0.01.

We then assessed the levels of inflammatory cytokines including TNF-α, IFN-γ and IL-6 levels in serum of mice. Con A resulted in increased production of IFN-γ, TNF-α and IL-6 in non-treated mice, which peaked at 8, 4, and 8 hours, respectively (Fig. 4A), while galectin-9 pretreatment led to a significant decrease in IFN-γ, TNF-α and IL-6 levels in the serum (Fig. 5A). To investigate whether galectin-9 directly inhibits cytokine release, spleen macrophages were incubated with 100 ng/mL LPS or LPS with galectin-9. As shown in Fig. 5B, adding galectin-9 markedly reduced LPS-induced release of IFN-γ, TNF-α and IL-6, indicating that galectin-9 pretreatment efficiently impaired proinflammatory immune responses by significantly reducing proinflammatory cytokines production. This effect may ameliorate Con A-induced hepatitis.

### Galectin-9 Induces Apoptosis of Activated CD4^+^ T Cells Both *in vivo* and *in* *vitro*

**Figure 6:**
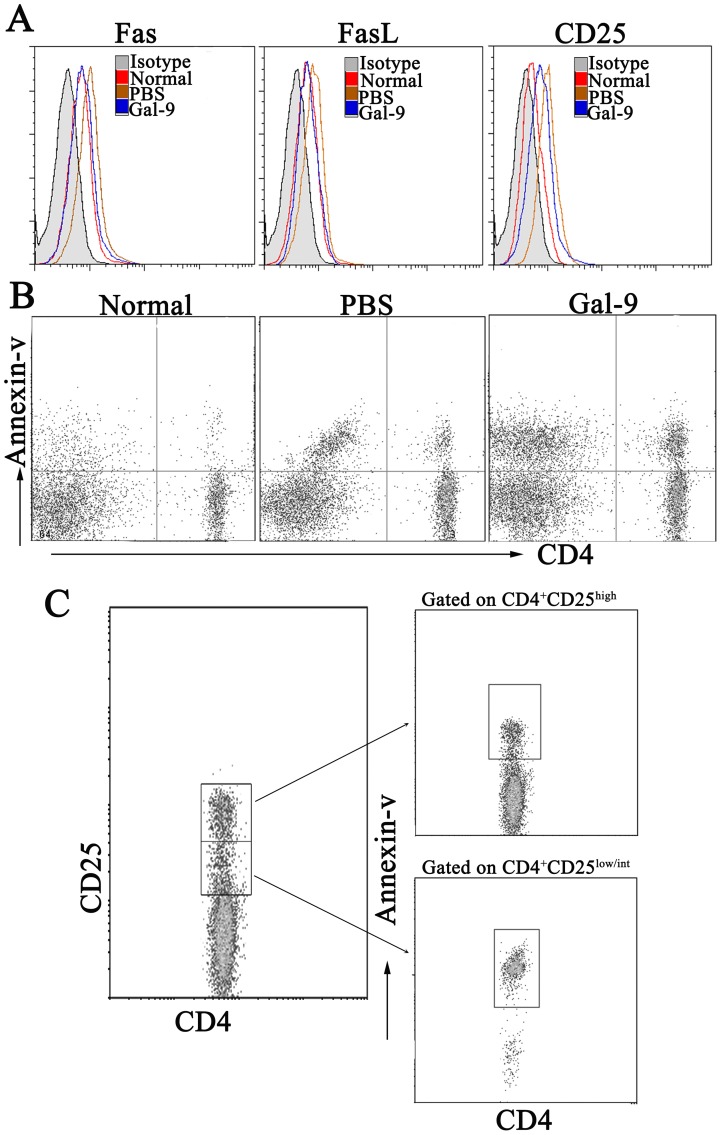
Galectin-9 induced apoptosis of CD4^+^ T cells *in vivo*. Galectin-9 (100 µg per mouse) or PBS was administrated i.v. to Balb/c mice (n = 8 per group) 30 min before Con A injection (20 mg kg^–1^). The splenocytes were isolated 24 h later. (A) Fas, FasL and CD25 expression on CD4^+^ T cells are shown. (B) Apoptosis of CD4^+^ T cells was analyzed by FACS. (C) Differentially induced apoptosis of CD4^+^ effector T cells and regulatory T cells by galectin-9 in Con A treated mice. Similar results were obtained in 3 separate experiments and the representative results were shown. Normal, normal mice; PBS, PBS pretreatment in Con A-treated mice; Gal-9, galectin-9 pretreatment in Con A-treated mice.

As shown in Fig. 6A, the expression levels of Fas, FasL and CD25 were low in CD4^+^ T cells from the spleen of normal mice. Con A administration significantly increased the percentage of Fas, FasL and CD25 expressing CD4^+^ T cells, and galectin-9 pretreatment prevented the increase.

In the model of Con A-induced hepatitis, since inflammation appeared to be mainly orchestrated by IFN-γ producing CD4^+^ T cells, we investigated the effects of galectin-9 on the induction of apoptosis of CD4^+^ T cells. As shown in results Fig.6B, about 50% CD4^+^ T cells were apoptotic after galectin-9 pretreatment, perhaps accounting in part for the anti-inflammatory effect of galectin-9. Furthermore, while about 90% of CD4^+^CD25^low/int^ effector T cells were apoptotic, only 10% of CD4^+^CD25^high^ Tregs were apoptotic by annexin V^+^ staining. These results indicated that galectin-9 administration efficiently down-regulates CD4^+^ effector T cell responses.

**Figure 7:**
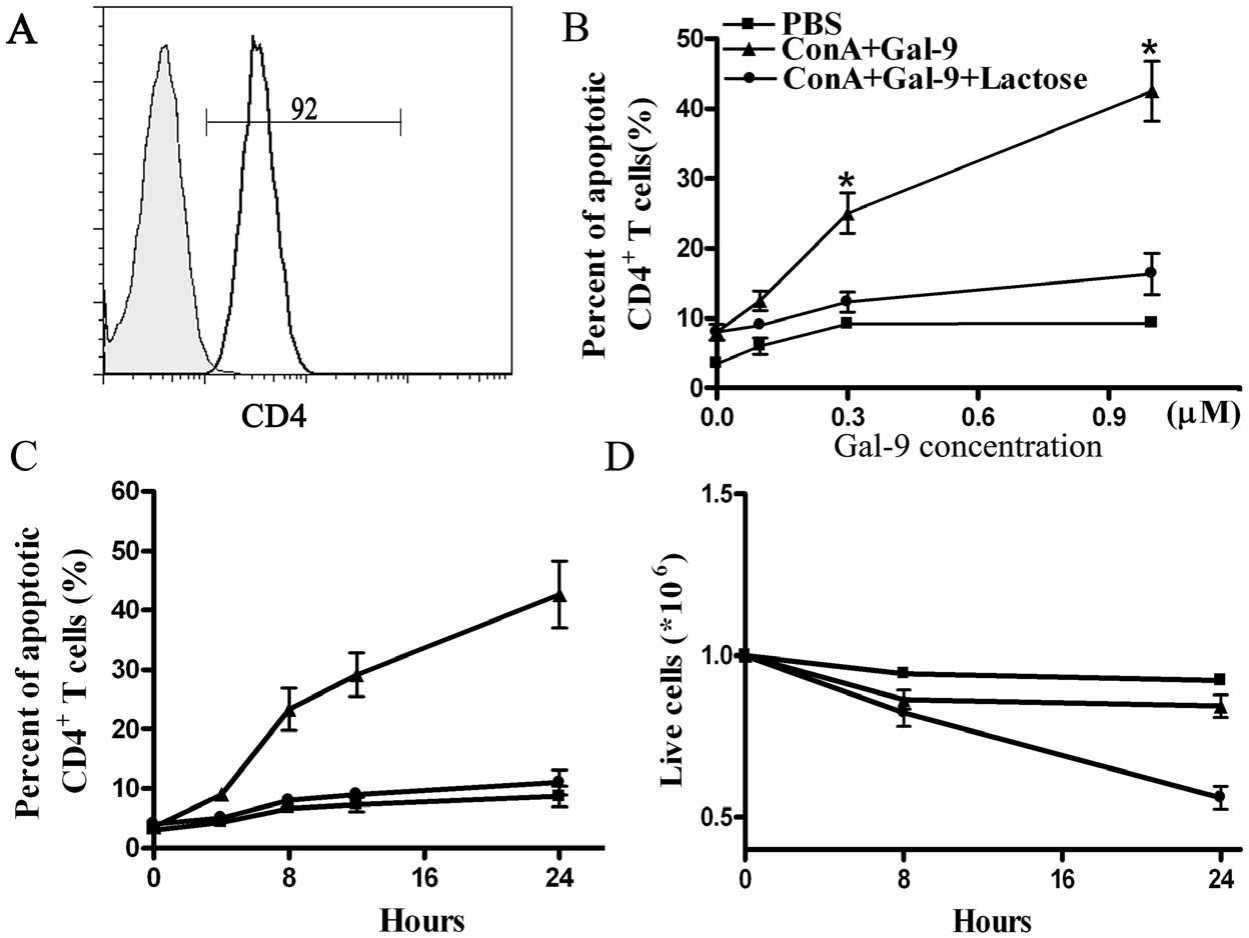
Galectin-9 induced apoptosis of Con A–activated CD4^+^ T cells at day-5. (A) The purity of isolated CD4^+^ T cells. (B) Spleen CD4^+^ T cells, isolated from untreated mice, were cultured with 10 µg/mL Con A for 5 days and then cultured for 24 hours at 37°C with or without 30 mM lactose followed by indicated doses of galectin-9, then apoptotic cells were assessed by FACS. (C) Time-course of galectin-9 (1 µM) induced apoptosis of day-5 Con A–activated T cells. (D) Absolute number of live cells after galectin-9 treatment, determined by Trypan blue dye exclusion. The results were presented as the mean ± SD of three separate experiments. *, P<0.05 vs PBS treatment.

We then assessed *in vitro* whether galectin-9 induces apoptosis in activated CD4^+^ T lymphocytes. Spleen CD4^+^ T cells, sorted from untreated mice (Fig. 7A), were activated by 5 days incubation with 10 µg/mL Con A and then challenged with several doses of galectin-9. Fig. 7B–D shows that galectin-9 increased the level of apoptosis in Con A–activated CD4^+^ T cells in a dose- and time-dependent manner, but had no effect on resting CD4^+^ T lymphocytes. Galectin-9–induced cell death was reversed by adding 30 mM lactose, indicating that β-galactoside binding activity is required for galectin-9-induced apoptosis.

## Discussion

The Con A–induced hepatitis, a T-cell–dependent model of liver damage, is regarded as an appropriate model of human immune-mediated liver disease. T cell activation plays a crucial role in the process of Con A–induced hepatitis, because severe combined immunodeficiency disorder mice, which lack mature T cells, are resistant to the damage induced by this plant mitogen [5], [6]. In this model of liver injury, there are at least 2 partially independent pathways by which activated T cells cause liver cell death. Cell death is induced either from the release of Th1-like cytokines such as TNF-α and IFN-γ, or by activation of the Fas/FasL apoptotic pathway on the hepatocyte cell surface. Indeed, earlier studies have shown that TNF-α or IFN-γ immuno-neutralization or gene ablation [9]–[12] and Fas or FasL gene deficiency [26], [27] confer protection against Con A–induced liver damage. In the present study we confirmed that CD4^+^ T-cell activation plays a prominent role in this model of liver injury. Depletion of CD4^+^ T cells reduced liver injury. These data consist with a recent manuscript showing that CD4 depletion reduces the clinical signs of Con A-induce hepatitis [28]. Moreover, Con A injection rapidly up-regulated CD25, Fas, and FasL which are activation-induced membrane antigens on peripheral CD4^+^ T cells, and this event was associated with severe CD4^+^ T lymphocyte infiltration of the liver.

Galectin-9 is a ligand for TIM-3 that is expressed on the surface of both Th17 cells and Th1 cells, and these cell types are critically involved in initiation of inflammatory and autoimmune disease [17], [18]. Zhu *et al.* revealed that TIM-3–galectin-9 pathway has evolved to ensure effective termination of effector Th1 cells [19]. Thus, it is possible that galectin-9 plays a role in the pathology of Con A–induced hepatitis by regulating effector Th1 cells. In the present study, we provided experimental evidence of an *in vivo* therapeutic role for galectin-9 in a murine model of T cell-mediated liver injury. Indeed, biochemical and histopathological data indicated that a single injection of galectin-9 was sufficient to protect against Con A–induced liver failure. In an attempt to elucidate how galectin-9 exerts this protective effect, we found that pretreating mice with galectin-9 almost completely prevented peripheral CD4^+^ T cell activation and infiltration induced by Con A. In particular, flow cytometry showed a marked reduction in the percentage of FasL-positive T cells in galectin-9–pretreated mice. This effect, together with the prevention of liver cell infiltration observed from histological analysis, is consistent with a previous finding that Con A–activated FasL-bearing T cells can cause direct hepatocyte apoptosis by triggering the Fas receptor expressed on the hepatocyte cell surface [26], [27]. The reduction in the percentage of activated CD4^+^ T cells observed in galectin-9–pretreated mice could be caused either by the prevention of CD4^+^ T-cell activation induced by Con A, as suggested by the observation that galectin-9 induced elimination of Con A–activated CD4^+^ T cells. In support of this, we found more apoptotic CD4^+^ T cells in galectin-9–pretreated mice than in mice treated with Con A alone. Moreover, we found that CD4^+^CD25^low/int^ effector T cells in galectin-9–pretreated mice were more susceptible to apoptosis, and that the Tregs-to-effector T cell ratio was increased, perhaps accounting in part for the anti-inflammatory effect of galectin-9. In addition, galectin-9 also caused selective apoptosis of Con A–activated CD4^+^ T cells *in vitro*, although it was ineffective on resting CD4^+^ T cells. These observations concur with the *in vitro* evidence that galectin-9 drives activated CD4^+^ T cells to apoptosis by signaling through TIM-3 [19].

In addition to its direct effect on activated CD4^+^ T cells, we found that galectin-9 also prevented the Con A–induced serum release of TNF-α, IL-6 and IFN-γ. This observation is of relevance because TNF-α and IFN-γ cause a direct and synergistic cytotoxic effect on hepatocytes *in vitro* [29], [30]. Although the proapoptotic activity of galectin-9 on Th1 lymphocytes could explain the inhibitory effect we observed in cytokine release, we showed that galectin-9 prevented LPS-induced cytokine release also from spleen macrophages *in vitro*. These data suggest that inhibition of proinflammatory cytokines represents an alternative inhibitory mechanism for explaining the suppressive properties of galectin-9 on T-cell–mediated diseases.

Taken together, our results are consistent with the observation that the TIM-3/galectin-9 interaction plays a critical role in Con A–induced hepatitis. This is further supported by the observation that hepatitis became more severe if signals from endogenous galectin-9 were blocked with anti-TIM-3 mAb. The TIM-3/galectin-9 interaction can be a potential therapeutic target given that pretreatment with galectin-9 protected mice from Con A–induced hepatitis. Our observations can be explained by multiple mechanisms, but one possibility is that the protective effect of galectin-9 involves a selective elimination of activated CD4^+^ effector T cells as well as prevention of synthesis and/or release of proinflammatory cytokines. Since galectin-9 is neither immunogenic nor cytotoxic, it could potentially be useful for therapy in human diseases involving T-cell activation.

## Materials and Methods

### Ethics Statement

This study was carried out in strict accordance with the recommendations in the Guide for the Care and Use of Medical Laboratory Animals (Ministry of Health, P. R. China, 1998). The protocol was approved by the Medical Laboratory Animal Care and Use Committee of Anhui Province (Permit Number: 2009-0132) as well as the Ethical Committee of Yijishan hospital of Wannan Medical College (Permit Number: 20090015).

### Mice and Treatment

BALB/c (H-2^d^) mice (age 6–8 weeks, 25–30 g) were purchased from the Experimental Animal Center of Qinglongshan (Nanjing, P. R. China). Hepatic damage was induced by i.v. injection of Con A (20 mg kg^–1^) dissolved in pyrogen-free saline. Recombinant galectin-9 was diluted with pyrogen-free saline and injected in a single dose (100 ug per mice) intravenously (i.v.) 30 minutes before Con A. In the CD4 or CD8 mAb pretreatment group, control rat IgG2a or IgG2b (R&D, USA), CD4 (Clone: GK1.5; R&D, USA) and/or CD8 mAbs (Clone: 53–6.7; R&D, USA) (100 µg per mouse) was administrated i.p. to Balb/c mice 24 h before Con A injection (20 mg kg^–1^). In the TIM-3 mAb pretreatment group, mice were treated *in vivo* by i.v. injection with 100 µg of neutralizing anti-TIM-3 (Clone: RMT3-23, Bio-X-cell) or control rat IgG2a mAb (BD Biosciences, CA) at 30 min before Con A administration.

### Transaminase Plasma Activities

Mice were killed 24 hours after Con A injection and blood samples were collected into 2 mL heparinized tubes. After centrifugation, plasma was recovered and immediately frozen at −80°C. Plasma alanine transaminase (ALT) and aspartate transaminase (AST) activities were measured by a 7170 Hitachi automatic analyzer (Hitachi, Japan).

### Histology Analysis

Livers from individual mice were cut longitudinally, fixed in 10% phosphate-buffered formalin and embedded in paraffin. Sections 5 µm thick were cut at various depths in the tissue section and stained with H&E to determine the level of inflammation. Each section was examined for evidence of mononuclear and polymorphonuclear cellular infiltration and necrosis. Histologic examination was done without the knowledge of the treatment given. Sections were examined by two independent investigators in a blind manner.

### Detection of Liver DNA Fragmentation

We measureed the extent of liver DNA fragmention using a cell detection enzyme-linked immunosorbent assay (ELISA) kit (Boehringer Mannheim AG, Switzerland) according to the manufacturer’s instructions. This assay is designed to quantify cytosolic oligonucleosome-bound DNA (histone ELISA). The procedure was adapted for liver tissue fragments as previously reported [31], [32]. Briefly, small pieces of liver (25–50 mg) were weighed and homogenized in lysis buffer, incubated for 30 minutes at room temperature, and, after 10 minutes of centrifugation at 2,000 rpm, 20 µL of the supernatant was tested with the ELISA kit.

### Measurement of Liver Caspase 3–like Activity

24 mice were killed 4, 8, 12, and 24 hours after Con A administration, and caspase3–like activity was measured in liver homogenates using 7-amino-4-trifluoromethyl coumarin (AFC)-DEVD as fluorescent substrate (Apo-Alert CPP32, Clontech Laboratories, Palo Alto, CA). Proteolytic cleavage was assessed at an excitation wavelength of 380 nm and an emission wavelength of 460 nm. Specificity for caspase 3–like enzymatic activity was shown by inhibition with 10 nmol/L DEVD-CHO. Each sample was assayed at 37°C in a modified thermostat cuvette holder in 1.25 mL of 100 mmol/L HEPES, 10%sucrose, 0.1 CHAPS, 2 mmol/L DTT, at pH 7.5, in an Hitachi 2000 fluorimeter (Pabish, Milan, Italy). Calibration was performed with a standard solution of Ac-AFC.

### ELISA Assay of Inflammatory Cytokines

Serum TNF-α, IFN-γ and IL-6 levels were measured 0, 4, 8, 12, 16 and 24 hours after Con A administration. At these time points, mice were killed and blood was collected into 2 mL heparinized tubes. After centrifugation, serum was recovered and stored in aliquots at −80°C until assayed. To investigate the effect of galectin-9 on TNF-α, IFN-γ and IL-6 release *in vitro*, mice were killed, and spleens were collected and maintained in a sterile RPMI 1640 medium containing 0.5% glutamine and 0.5% sterile endotoxin-free FCS. The content of the spleens were collected in a Petri dish and diluted with RPMI 1640. After repeated washes, pellets were resuspended in complete medium and incubated at 37°C for 24 hours. Adherent macrophages were scraped from the flask and incubated (1×10^6^ cells/mL) for 24 hours with 100 ng/mL LPS, with or without galectin-9 (10 µg/mL) added at the same time as LPS. At the end of the incubation period, aliquots of the supernatant were collected and stored at −80°C until assayed. TNF-α, IFN-γ and IL-6 concentrations were measured with specific ELISA kits (R&D Systems, USA).

### FACS Analysis

Individual mononuclear cell suspensions were pooled from spleen. Cells were stained with the following mAbs (eBioscience Inc., USA) diluted in 1% FBS in PBS: TIM-3, CD4, CD25, Fas, and FasL. For intracellular staining, cells were fixed and permeabilized using fixation buffer and permeabilization solution or an anti-mouse Foxp3 staining kit (eBioscience Inc., USA) or anti-mouse IL-17/IFN-γ (BD Biosciences, CA). Cell fluorescence was measured using FACS and data analyzed using Cell Quest software (BD Biosciences, CA).

### Analysis of CD4^+^ T Cell Apoptosis *in vivo* and *in vitro*

Mice that were pretreated with or without galectin-9 were killed 24 hours after Con A administration. The spleens were removed and mononuclear cells were separated using lymphocyte separation medium. CD4^+^ T cell apoptosis was evaluated at the end of the isolation procedure. To investigate whether galectin-9 induces selective apoptosis of Con A–activated CD4^+^ T cells *in vitro*, CD4^+^ T cells were purified from naive BALB/c mice by magnetic cell sorting using CD4^+^ T cell isolation kits (Miltenyi Biotech) according to the manufacturer’s instructions. The purity of CD4^+^ T cells was >90% as assessed by FACS. Sorted CD4^+^ T cells were cultured with 10 µg/mL Con A in complete medium (RPMI 1640, 10 mmol/L HEPES, 10% FCS) at 37°C for 5 days. Day-5 blasts were then centrifuged over Ficoll-Paque to enrich for viable cells and were cultured for 24 hours at 37°C in complete medium with 3 mmol/L DTT with or without galectin-9, alone or in combination with 0.1 mol/L lactose to prevent galectin-9 binding to its receptor. At the end of the incubation period, cell apoptosis was measured. The proportion of apoptotic cells was determined by staining with annexin V and/or CD4, CD25. Stained cells were analyzed by FACS.

### Statistical Analysis

Data are shown as the mean ± SEM. Statistical analysis of the data was performed with the two-tailed independent Student's t-test or the ANOVA analysis using the GraphPad Prism (Version 4.0) statistical program. P<0.05 was considered statistically significant.
